# Simulation Study on Dielectric Constant Sensing by Interference of Spoof Surface Plasmon Polaritons

**DOI:** 10.3390/mi17050517

**Published:** 2026-04-24

**Authors:** Ting Zeng, Chunyang Bi, Jun Zhou, Sen Gong

**Affiliations:** 1 School of Materials and Energy, University of Electronic Science and Technology of China, Chengdu 611731, China; 2 School of Pharmaceutical Sciences, Chengdu Medical College, Chengdu 610500, China; 3 School of Electronics Science and Engineering, University of Electronic Science and Technology of China, Chengdu 611731, China

**Keywords:** spoof surface plasmon polaritons (SSPPs), dielectric constant variation detection, cutoff frequency, destructive interference, frequency shift

## Abstract

Detecting changes in the permittivities of materials has important applications in electronic information, materials science, biomedicine, and many other fields. However, existing detection methods are limited by factors such as sample thickness and resonance intensity, making it difficult to achieve sensitive dielectric constant detection at desired frequency bands. This paper proposes a method for detecting the dielectric constant changes in samples based on destructive interference of spoof surface plasmon polaritons (SSPPs) in a dual-path transmission structure, which forms a characteristic absorption peak at the SSPPs’ cutoff frequency. Specifically, by utilizing the dependence of the SSPPs’ phase on the periodic unit, a constant π phase difference is formed at the cutoff frequency through the periodic unit number difference between the two paths, resulting in a cutoff frequency absorption peak. When the sample is coated on the SSPPs’ dual-path structure, the boundary conditions are altered, leading to a cutoff frequency shift, thereby enabling dielectric constant detection at the specified frequency. Simulation results show that, with proper structural design, the normalized characteristic frequency shift reaches 10.8%/$\varepsilon_{S}$ and further demonstrates dramatic robustness against initial phase difference, sample thickness and sample loss. In summary, this work provides a novel high-precision and high-robustness method for detecting dielectric constant changes in samples at specified frequencies.

## 1. Introduction

The dielectric constant is one of the core electromagnetic properties of materials. Highly sensitive detection of its variations is crucial for the development of key topics, such as microwave and radio frequency device design [1,2], functional material evaluation [3,4,5], and biomedical detection [3,4,5], in numerous fields of electronic information, materials science, and biomedicine. Therefore, the development of high-sensitivity dielectric constant measurement technology holds significant scientific importance and application value.

With the development of microelectronics technology, chip-based sensors have demonstrated great potential in the field of dielectric constant variation measurement by virtue of their advantages of miniaturization, integration, low cost and convenience for mass production [6,7]. Their measurement principles are mainly based on two mechanisms. The first is the resonance principle. For example, sensors based on micro-ring resonators and dielectric resonators detect the variation in the dielectric constant of surrounding media by monitoring the shift in resonance frequency [8,9,10,11,12,13]. For example, Wei et al. integrated a microfluidic channel with a substrate integrated waveguide (SIW) reentrant cavity to achieve the measurement of the complex permittivity of liquids through an enhanced localized electric field [10], and Navaei et al. utilized a microstrip line coupled with a split-ring resonator (SRR) to characterize the dielectric constant of trace liquids through resonant frequency shift [12]. The second is the reflection/transmission principle. For instance, sensors based on microstrip lines and coplanar waveguides (CPWs) extract dielectric information by analyzing the amplitude and phase variations of electromagnetic waves during transmission [2,14,15,16,17]. For example, Bergmann et al. achieved the extraction of the dielectric constant of fused silica in the range up to 325 GHz based on an on-chip coplanar CPW transmission line combined with mTRL calibration [2], and Narayanan proposed a broadband dielectric constant measurement method based on the scattering parameters of microstrip transmission lines, which can reduce errors caused by connection repeatability [16].

Although chip-based sensors exhibit significant advantages in dielectric constant measurement, the existing technologies still face numerous challenges. For sensors based on the resonance principle, their measurement sensitivity highly depends on the quality factor (Q-factor) of the resonator. The intrinsic dielectric loss of the material, surface roughness during fabrication, and loss introduced by packaging significantly reduces the Q-factor, thus limiting the measurement accuracy [6,7]. In addition, the resonant frequency is susceptible to temperature drift and environmental perturbations, which necessitates complex temperature compensation and stabilization schemes [6,18]. For sensors based on the reflection/transmission principle, the measurement results are extremely sensitive to the thickness, flatness and surface properties of the sample. Furthermore, the inherent dispersion characteristics of the material lead to frequency dependence of the measurement results, which usually requires complex numerical algorithms (e.g., the Nicolson–Ross–Weir algorithm) for inversion and correction [3,4,5] and thus increases the system complexity. Therefore, it is urgent to explore a high-sensitivity dielectric constant sensing method that is robust to sample size, material dispersion and fabrication errors.

Spoof surface plasmon polaritons (SSPPs), as a novel electromagnetic mode supported by artificially designed periodic metallic structures, offer a new solution to the challenges mentioned above by their distinct advantages [19,20,21,22,23,24,25,26,27]. First, SSPPs can strongly confine electromagnetic fields to the subwavelength scale near the surface of metallic structures [28,29,30,31], thereby controlling the field–material interaction area [19,20,21] and enhancing the resonance Q-factor [22,23,24]. Second, the dispersion characteristics of SSPPs are mainly determined by the geometric parameters of the structures [28,29,32], while the dielectric properties of the sample only act as a perturbation to modify their dispersion curves [33,34], which endows the sensor with a certain robustness against the intrinsic material dispersion of the sample. Most importantly, the cutoff frequency wave vector of SSPPs, determined by their periodic structural design [29,32], is independent of the dielectric constant of the sample, which provides a physical foundation for achieving robust measurement of dielectric constant variations.

Based on the above analysis, this paper presents a dielectric constant sensing method utilizing the interference effect of two-path SSPPs. By exploiting the distinctive wave vector characteristics of SSPPs at the cutoff frequency, a characteristic absorption peak sensitive to sample permittivity variation is established, enabling high-sensitivity and high-robustness dielectric constant measurement. Specifically, below the cutoff frequency, interference between two SSPP paths induced by their phase difference gives rise to absorption that increases with frequency. Above the cutoff frequency, the absorption drops sharply due to the transmission stopband characteristic of SSPPs. In this way, a characteristic absorption peak dependent on the cutoff frequency is formed. By monitoring the frequency shift in this characteristic absorption peak, the variation range and trend of the sample dielectric constant can be directly retrieved. Following this principle, we design and simulate an on-chip dielectric constant sensor operating in the D-band (110–170 GHz). The simulation results show that with proper structural design, the sensor exhibits excellent performance: the normalized characteristic frequency shift reaches as high as 10.8%/$\varepsilon_{S}$, demonstrating ultra-high sensitivity; meanwhile, the tolerance analyses of the initial phase difference, sample thickness and sample loss also demonstrate the strong test robustness of this structure.

The rest of this paper is organized as follows. First, the fundamental principle of SSPP interference and the physical mechanism of the characteristic absorption peak formed at the cutoff frequency are elaborated in detail. Second, the formation process of the characteristic absorption peak at the cutoff frequency is systematically investigated via simulation, and the effects of the sample dielectric constant variation on the frequency and intensity of the absorption peak are quantitatively analyzed. Finally, the main error sources in the interference process are analyzed in depth, so as to verify the robustness of the proposed method.

## 2. Principle of Permittivity Variation Detection by SSPP Cutoff Frequency

**Figure 1:**
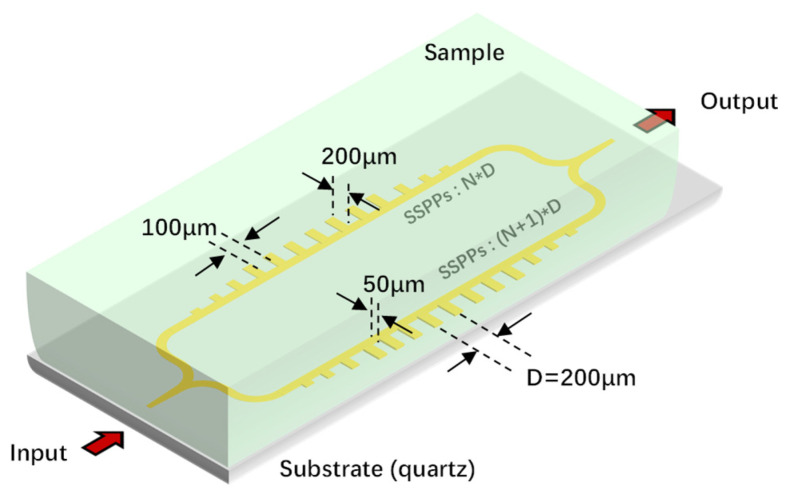
Schematic diagram of the structure: The structure comprises two identical periodic SSPP transmission lines (period D) on a quartz substrate with different numbers of periodic units, and the sample under test is placed on the chip surface.

The structure is shown in Figure 1, which adopts the principle of two-path interference to measure the tiny changes in the dielectric constant of the sample. Specifically, the input signal is respectively input into two SSPP transmission lines with a fixed periodic unit number difference through a 3 dB power divider, and then output after being combined by a combiner. The sample to be tested is placed on the chip surface and covers both optical paths simultaneously. This process can be simply expressed as

(1) $$A=A_{1}e^{-jk_{1}L_{1}}+A_{1}e^{-jk_{2}L_{2}}$$

where $A_{1}$ and $A_{2}$ are the amplitudes of the two SSPP waves, $L_{1}$ and $L_{2}$ are the optical paths of the signals, and $k_{1}$ and $k_{2}$ are the corresponding wave vectors. At this time, $k_{1}$ is equal to $k_{2}$. It can be seen from Equation (1) that when the phase difference between the two paths is

(2) $$k_{1}\left(L_{1}-L_{2}\right)=(2n+1)\pi$$

the interferometric synthetic field intensity is zero, forming an absorption peak.

For a fixed path difference, the zero-frequency of the synthetic S_21_ is directly determined by the relationship between the wave vector k and the frequency, which is the dispersion relation. When the sample to be measured is coated on the surface of the circuit, the tiny change in the dielectric constant of the sample leads to a change in the dispersions of the two transmission lines, which causes the frequency to change at the π × n phase difference. Thus, the change in the dielectric constant of the sample can be characterized by the frequency change in the zero point of the interferometric synthetic wave S_21_.

**Figure 2:**
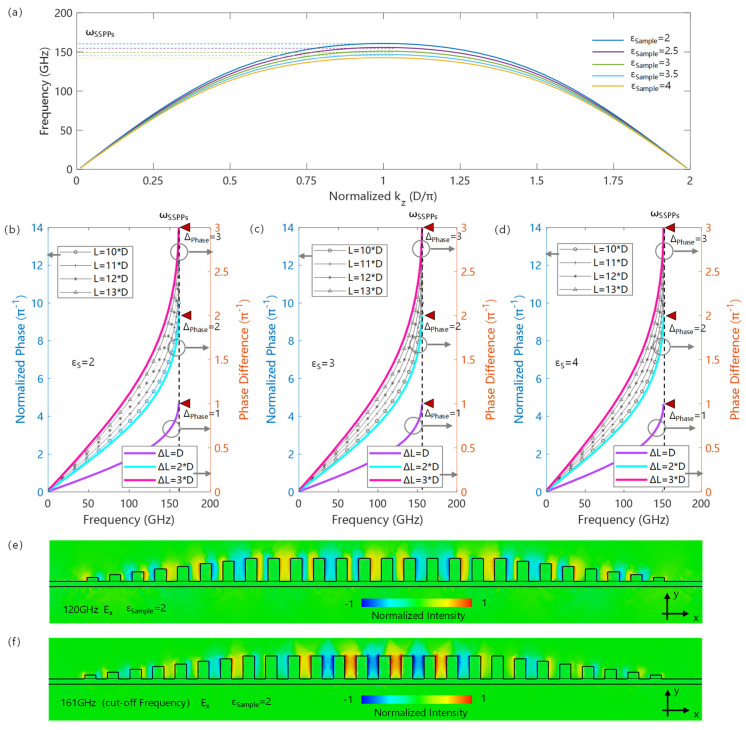
Principle of constant phase difference in SSPP transmission lines: (**a**) SSPP dispersion curves for different sample permittivities; (**b**–**d**) show the relationship between the phase and frequency for different sample permittivities and period unit numbers, and the phase difference corresponds to the right vertical axis, while the phase corresponds to the left vertical axis; (**e**) field distribution in the SSPP transmission line below the cutoff frequency (120 GHz); (**f**) field distribution in the SSPP transmission line at the cutoff frequency (161 GHz).

Figure 2a shows the dispersion relation of the SSPP transmission line in the chip structure, with a period D of 200 μm, a slot depth of 200 μm, a duty cycle of 0.5, a line width of 50 μm and a substrate thickness of 50 μm. The microstrip input and output of the SSPP transmission line adopt a slot depth gradient coupling structure. And the structure is studied by the simulation method of finite-difference time-domain (FDTD). Since SSPPs originate from the Floquet boundary conditions provided by the periodically arranged microstructures, their dispersion curves have typical periodic characteristics. As shown in Figure 2a, with the increase in the wave vector, the corresponding frequency begins to increase; however, as the wave vector further increases, the SSPPs exhibit a cutoff frequency $\omega_{SSPPs}$ determined by the structural characteristics and boundary conditions. At the cutoff frequency, the wave vector is only determined by the period of the structure and has nothing to do with the specific value of the cutoff frequency. According to Floquet’s theorem, the cutoff frequency has a wave vector determined by the structure:

(3) $$k_{\omega p}=\pi /D$$

Meanwhile, the value of the cutoff frequency is only determined by the design of the periodic structural units and the constitutive relation of the surrounding medium. With the increase in the sample dielectric constant, the SSPP cutoff frequency decreases, while the corresponding wave vector remains unchanged, as shown by the dispersion curves corresponding to different media in Figure 2a.

According to the wave vector condition at the zero point of the synthetic wave in Equation (2) and the cutoff frequency wave vector in Equation (3), it can be further concluded that the path difference required for the destructive interference of the two SSPP waves must satisfy

(4) $$∆L=\left(2n+1\right)\times D$$

where n is an integer. Specifically, when the path difference between the two SSPP waves differs by an odd number of periods, the phase difference at the $\omega_{SSPPs}$ is strictly (2n + 1)π, which satisfies the zero-point condition of the synthetic wave.

Figure 2b–d illustrate the frequency–phase relationships under different boundary dielectric constants. It can be observed that at low frequencies, the phase difference increases linearly with increasing frequency, which is attributed to the SSPP mode exhibiting TEM-like transmission characteristics with weak dispersion, where the phase difference induced by fixed path accumulation is only frequency-dependent. As the frequency increases, the dispersion of SSPPs becomes more pronounced, leading to a certain correlation between the phase difference and the structure. At the cutoff frequency, the phase difference is directly determined by the difference *dN* in the number of periodic units, i.e., *dN* × π.

Meanwhile, different boundary dielectric constants show similar properties: all exhibit a TEM-like phase difference relationship in the low-frequency band and an integer multiple of π at their corresponding cutoff frequencies. This is because the dispersion characteristics of SSPPs are mainly determined by the periodic structure, and the transmission of SSPP modes in the strong dispersion region is dominated by the coupling between structural units, resulting in the phase difference being related to the number of periodic units.

This phenomenon can be further confirmed by the field distribution diagrams shown in Figure 2e,f. As illustrated in Figure 2e, below the cutoff frequency, the field distribution of the SSPP mode spans multiple periodic units, and the phase difference between different structural units exhibits a quasi-continuous distribution characteristic. Thus, the phase accumulation is mainly derived from the accumulation of the optical path and has no direct relationship with the number of periods. As the frequency increases to the cutoff frequency, as shown in Figure 2f, the field distribution of the SSPP mode is closely related to the structural characteristics, and its transmission process originates from the coupling between periodic units. At this time, the phase accumulation comes from the increase in the number of couplings, leading to a close relationship with the number of periods rather than the transmission distance. At this point, the field directions between the two adjacent periods are out of phase, resulting in a strict 180° phase difference. In addition, due to the fact that such a phase difference originates from the periodic characteristics of the structure, different boundary dielectric constants only change the cutoff frequency, so that under different sample coverages, the SSPP waves with an odd number of unit structure differences all exhibit a phase difference of an integer multiple of π. Therefore, for a fixed periodic structure, the variation in the sample permittivity only induces a corresponding change in the dispersion relation, which is further manifested in the cutoff frequency shift. Combined with the invariant phase difference, the permittivity variation introduced by the sample can be directly characterized.

## 3. Characteristic Absorption Spectra and Tolerance of Permittivity Variation

**Figure 3:**
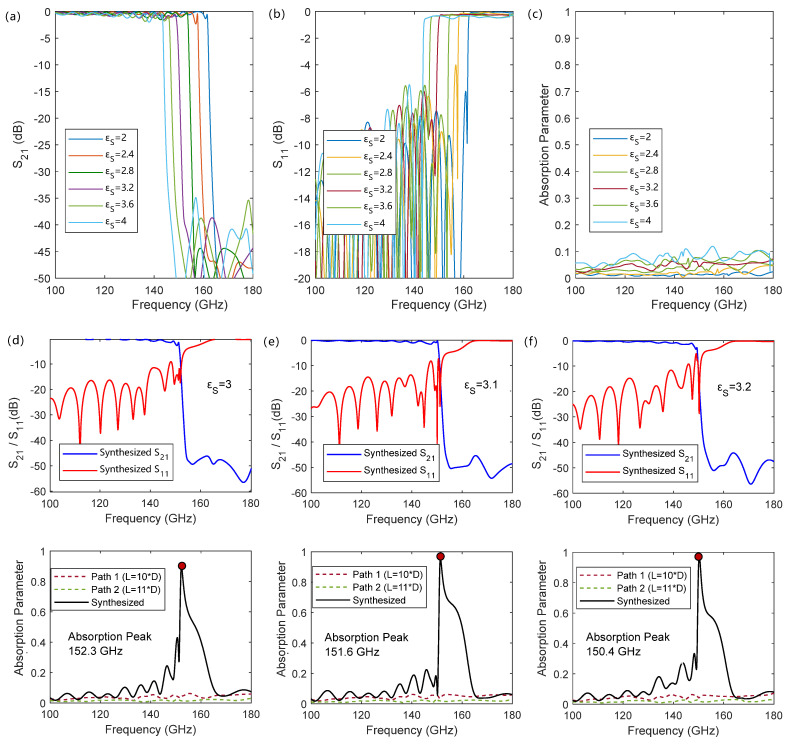
Transmission and absorption characteristics of single-path SSPPs and after interference of dual-path SSPPs for the sample thickness of 500 μm: (**a**–**c**) show the S-parameter and absorption characteristics of a single-path SSPP transmission line, and more data can be found in Figure A1; (**d**–**f**) show the transmission and absorption characteristics synthesized by two paths of SSPPs with a period unit number difference of 1.

Figure 3 shows the S-parameter curves and absorption peaks of the one-path and two-path interference synthesis SSPPs for the sample thickness of 500 μm. As shown in Figure 3a, for a single-path SSPP, before the cutoff frequency $\omega_{SSPPs}$, its transmission characteristic S_21_ approaches 1, indicating that electromagnetic waves propagate along the SSPP structure; however, as the frequency exceeds the cutoff frequency, the transmission coefficient S_21_ decreases sharply, and the corresponding reflection coefficient S_11_ increases sharply, as shown in Figure 3b. This is because when the input frequency exceeds the cutoff frequency, it enters the forbidden band, the SSPP mode cannot be excited, and the incident energy is almost completely reflected. Such characteristics originate from the dispersion characteristics of the SSPP transmission line, which do not change with the number of structural units composing the SSPP transmission line, and the transmission characteristics change at the cutoff frequency. However, such an inflection-point characteristic frequency is often difficult to directly determine through measurement. As shown in Figure 3c, an obvious characteristic absorption peak of a single path of SSPPs is also difficult to form due to the switching between transmission and reflection.

To this end, the cutoff frequency can be extracted through the absorption peak by synthesizing two SSPP transmission lines with an odd difference in the number of structural units. Figure 3d–f show the synthesized transmission and absorption characteristics. It can be seen that before the cutoff frequency, due to the phase difference between the two transmitted SSPPs, their combined field gradually forms a traveling–standing wave in the overall structure. As the frequency gradually approaches the SSPPs’ characteristic frequency, the phase difference begins to quickly approach 180°, causing the transmission curve to start decreasing. At the SSPPs’ cutoff frequency, the phase difference between the two SSPP waves is π, forming a pure standing wave. As the frequency further increases into the SSPPs’ cutoff region, the input energy is completely reflected. This process makes the combined wave exhibit an obvious absorption peak at the SSPPs’ cutoff frequency. Before the cutoff frequency, the absorption growth originates from the destructive interference that changes with the phase difference, resulting in the shape of the absorption spectrum being trigonometric function type with frequency; after the cutoff frequency, the input wave is directly reflected, making the spectrum after the peak decrease sharply.

**Figure 4:**
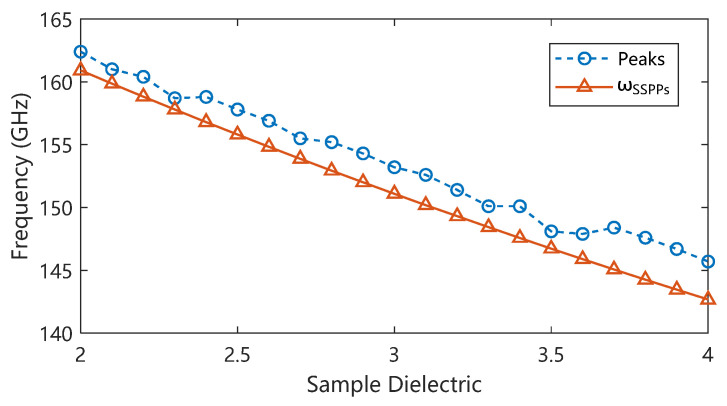
The dependence of the absorption characteristic peak frequency on permittivity.

**Figure A2:**
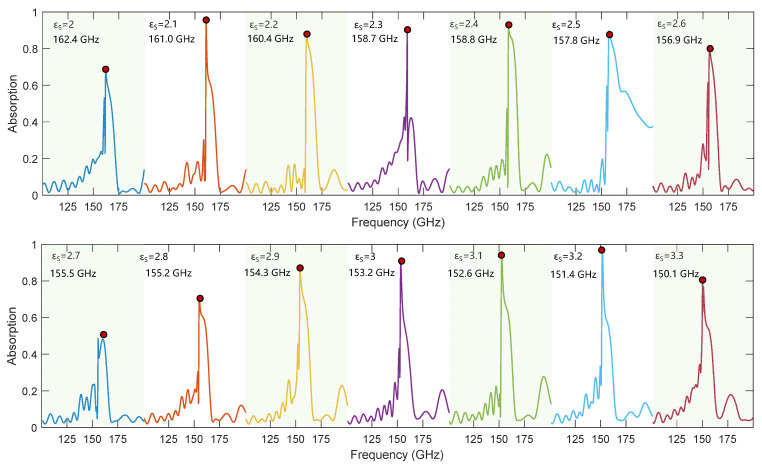
The interference absorption peaks for different sample dielectric constants.

Such absorption peak characteristics depend entirely on the dispersion characteristics of the structural SSPP transmission line. Changing the dielectric constant characteristics of the structural boundary causes the cutoff frequency to change, thereby changing the absorption peak frequency and further deriving the dielectric constant changes in the sample in the corresponding frequency band through the structural characteristics. The overall relationship between the sample dielectric constant and the absorption peak frequency is shown by the dashed dots in Figure 4: as the dielectric constant increases, the absorption peak frequency decreases, while a decrease in the sample dielectric constant leads to an increase in the absorption peak frequency. Benefiting from the change in the dispersion characteristics of SSPPs, when the dielectric constant of the sample changes from 2 to 4, the absorption characteristic frequency of the designed structure changes from 162.4 GHz to 145.7 GHz. The characteristic frequency shift caused by the unit change in the sample dielectric constant reaches 8.35 GHz, with a normalized unit frequency shift of 10.8%/$\varepsilon_{S}$ relative to the center frequency. As shown by the data comparison in Figure 4, the absorption characteristic frequency is higher than the expected cutoff frequency, and the simulation results show a certain fluctuation on both sides away from the designed detection center dielectric constant value of 3. This is caused by the additional resonance introduced by the reduced impedance matching degree of the input and output ports, which can be avoided by optimizing the design of the input and output coupling structures. Nevertheless, this trend is consistent with the dispersion characteristics of the SSPPs and shows an overall linear change. The absorption spectra for different dielectric constants can be found in Figure A2.

**Figure 5:**
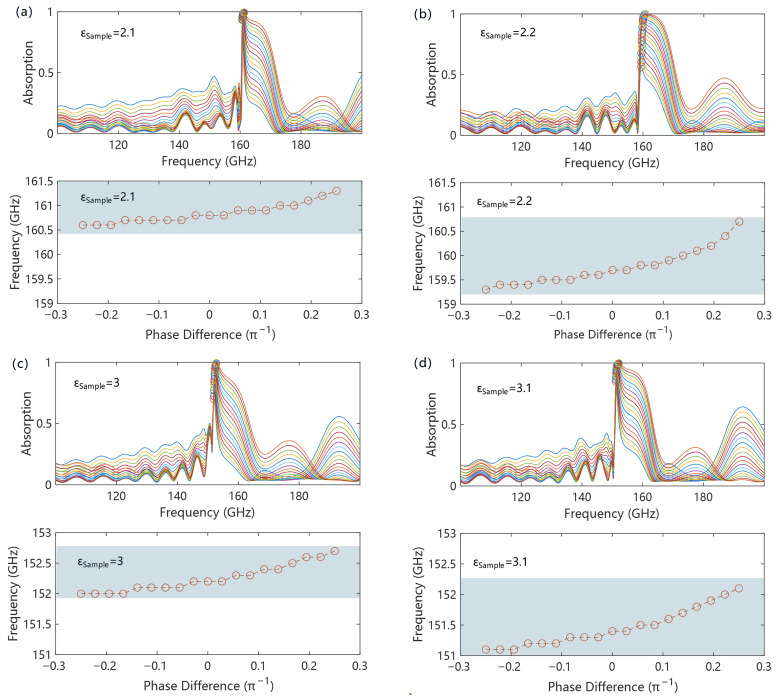
Initial phase difference tolerance of SSPP interference synthesis: (**a**–**d**) show the results of initial phase difference tolerance for different sample permittivities.

Based on this mechanism, the initial phase difference between the two transmission lines caused by the power divider, transmission coupling input–output resonance, and other factors is one of the main sources of test errors. Figure 5 shows the dependence of the absorption peak position on the initial phase difference under different sample dielectric constants. It can be found that the difference in initial phase leads to a change in the absorption peak position, which is because the initial phase difference affects the wave vector at the interference point, resulting in a frequency shift. However, benefiting from the strong dispersion characteristics of SSPPs near the cutoff frequency, a drastic change in wave vector only causes a slight frequency shift. This makes the influence of the initial phase difference on the absorption peak frequency not obvious. As shown in Figure 5a–d, within an initial phase difference of ±π/4, only an absorption frequency difference within 1.5 GHz is caused, and the shift rate is better than 1% of the center frequency, indicating good robustness against initial phase differences. Furthermore, for different sample dielectric constants, the absorption frequency shows the same shift direction and a peak shift amount equivalent to that within the dielectric constant test range, which makes it possible to correct the frequency shift caused by the initial phase difference through algorithms.

**Figure 6:**
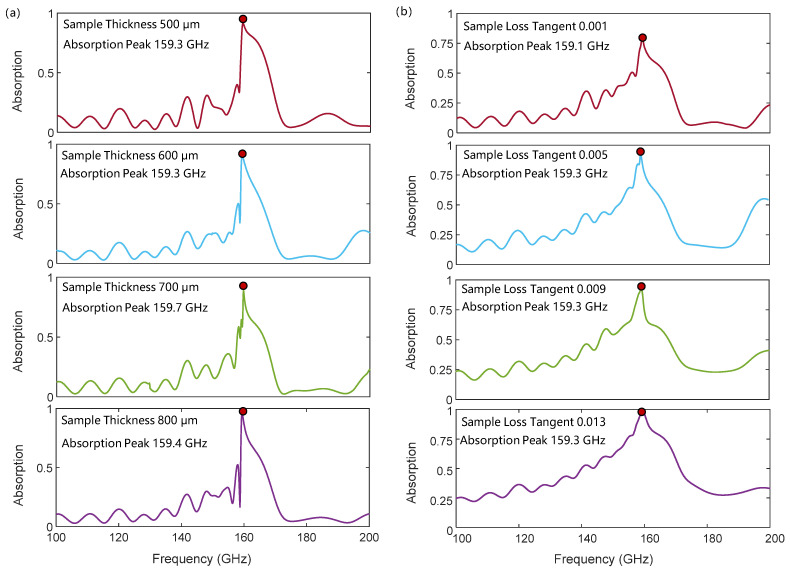
The influence of sample thickness (**a**) and loss (**b**) on the interference absorption peak.

Figure 6a shows the robustness of the characteristic absorption frequency with respect to sample thickness. Since SSPPs are typical surface waves, their fields are mainly confined near the metallic periodic structure and decay exponentially in the dielectrics on both sides. Therefore, when the thickness of the sample exceeds the penetration depth of SSPPs, the dispersion of SSPPs tends to stabilize, and the frequency of the interference absorption peak in this work also tends to be stable. As shown in Figure 6a, when the sample thickness varies from 500 μm to 800 μm, the characteristic absorption peak remains fixed near 159.4 GHz, and the frequency shift induced by the thickness variation is within 0.2%.

Figure 6b illustrates the influence of material loss on the characteristic absorption peak. It can be observed that the sample loss has little effect on the frequency of the characteristic absorption peak. When the loss tangent of the sample varies from 0.001 to 0.019, the corresponding absorption peak frequency ranges from 159.1 GHz to 159.3 GHz, with a frequency shift within 0.1%. This is because the frequency of the characteristic absorption peak is mainly determined by the phase difference between the two optical paths. However, the material loss induces identical dispersion corrections in both optical paths, so the phase difference between the two paths hardly changes with loss, making the absorption frequency insensitive to loss. The main effect of loss is reflected in the peak intensity, as shown in Figure 6b. Higher loss leads to greater attenuation of the incident wave during SSPP propagation, creating a stronger characteristic absorption peak.

## 4. Conclusions

To summarize, this paper proposes a material dielectric constant detection method based on the combination of SSPP cutoff frequency and destructive interference. The change in the sample’s dielectric constant causes a variation in the cutoff frequency of the SSPPs’ transmission line boundary conditions. A constant π phase difference is then formed at the cutoff frequency due to the difference in the number of periodic units in the SSPP transmission line. Finally, through two-path destructive interference, a characteristic absorption peak with a specific spectral shape is formed at the SSPPs’ cutoff frequency. Benefiting from the phase characteristics of the SSPP transmission mode, this characteristic absorption peak corresponds one-to-one with the SSPPs’ cutoff frequency, allowing for the inversion of the sample’s dielectric constant change through the corresponding boundary conditions. Simulation results show that, with proper design, the characteristic frequency shift rate induced by a unit change in the dielectric constant normalized to the designed center frequency reaches 10.8%/$\varepsilon_{S}$ in the D-band. Meanwhile, due to the localized nature and dispersion characteristics of the SSPP transmission mode, this scheme also demonstrates excellent robustness against test error sources such as initial phase differences, sample thickness and sample loss. This mechanism further indicates that the highly sensitive detection of the sample’s dielectric constant stems from the perturbation of the boundary conditions through minute changes, while its robustness originates from the intrinsic mode dispersion characteristics determined by the periodic structure. Therefore, this method can be further extended to a wide frequency range from microwaves to terahertz through different periodic structure designs. In conclusion, this paper presents a highly sensitive and environmentally robust method for measuring changes in a sample’s dielectric constant, applicable to multiple frequency bands.

## Figures and Tables

**Figure A1:**
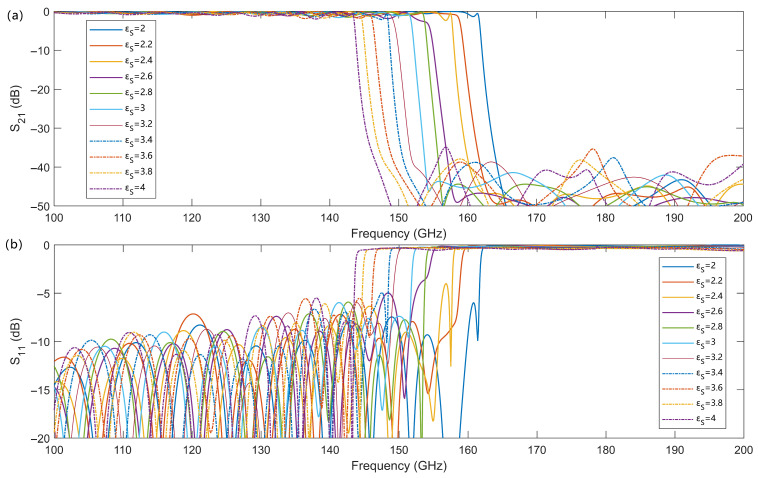
Transmission (**a**) and reflection (**b**) characteristics of single-path SSPPs for different sample dielectric constants.

## Data Availability

All relevant information is contained within the manuscript.

## Abbreviations

The following abbreviations are used in this manuscript:

SSPPs	Spoof surface plasmon polaritons
SIW	substrate integrated waveguide
SRR	split-ring resonator
CPW	coplanar waveguide
